# Palonosetron versus ondansetron as rescue medication for postoperative nausea and vomiting: a randomized, multicenter, open-label study

**DOI:** 10.1186/2050-6511-15-45

**Published:** 2014-08-16

**Authors:** Keith A Candiotti, Syed Raza Ahmed, David Cox, Tong J Gan

**Affiliations:** 1 University of Miami–Jackson Memorial Hospital, 1611 NW 12th Avenue, Room 300, 33136 Miami, FL, USA; 2 Becton, Dickinson and Company, 1 Becton Drive, 07417 Franklin Lakes, NJ, USA; 3 Eisai Inc., 100 Tice Boulevard, 07677 Woodcliff Lake, NJ, USA; 4 Duke University Medical Center, 2100 Erwin Road, 27710 Durham, NC, USA

**Keywords:** Postoperative nausea and vomiting, Antiemetics, Palonosetron, Ondansetron

## Abstract

**Background:**

This study compared palonosetron and ondansetron as rescue medications for postoperative nausea and vomiting (PONV) in patients who received prophylactic ondansetron. Although guidelines recommend use of an agent from a different class when prophylaxis has failed, palonosetron has unique properties relative to other serotonin 5-HT_3_ receptor antagonists. Prior trials assessing its use for rescue have had conflicting results. Although palonosetron has compared favorably with ondansetron for PONV prevention, the drugs have not been compared in the rescue setting of failure of 5-HT_3_ receptor antagonist prophylaxis.

**Methods:**

This was a randomized, open-label, multicenter trial comparing the efficacy and safety of intravenous palonosetron 0.075 mg and intravenous ondansetron 4 mg in patients experiencing PONV following laparoscopic abdominal or gynecological surgery despite prophylactic ondansetron.

**Results:**

Of 239 patients screened, 220 were enrolled and 98 were treated for PONV: 48 and 50 in the palonosetron and ondansetron arms, respectively. Complete control during 72 hours after study drug administration was achieved in 25.0% of palonosetron recipients and 18.0% of ondansetron recipients (95% confidence interval [CI], -9.2, 23.3; p = 0.40). Corresponding incidences of vomiting were 29.2% for palonosetron and 48.0% for ondansetron (95% CI, -0.06, 37.7; p = 0.057), and 62.5% and 56.0% required additional rescue treatment, respectively (95% CI, -25.9, 12.9; p = 0.52). Other than a similar incidence of procedural pain in the 2 groups, the most common treatment-emergent adverse events, which were generally mild, were headache (14.6% vs 12.0%), constipation (8.3% vs 10.0%), and dizziness (6.3% vs 8.0%), for the palonosetron and ondansetron groups, respectively.

**Conclusions:**

Palonosetron and ondansetron did not show differences in the primary efficacy endpoint of CC during the 72 hours after study drug administration. There was a trend toward less emesis in the 0–72 h time period favoring palonosetron. While larger studies are needed to fully assess any clinical benefits of palonosetron to rescue patients who have failed ondansetron prophylaxis for PONV, the benefit, if any, would be limited based on this study.

**Trial registration:**

ClinicalTrials.gov, NCT00967499 (Registered August 27, 2009)

## Background

Postoperative nausea and vomiting (PONV) is a frequent complication of surgery, with considerable medical and economic impact, and is associated with high levels of patient discomfort and dissatisfaction [1]. PONV is an especially distressing adverse event to many patients, often feared more than postoperative pain [1,2]. The incidence of PONV is estimated at 25% to 30% in all patients and as high as 80% in patients with multiple high-risk factors [3,4].

PONV, alone or combined with pain, is one of the leading causes for delayed discharge or unplanned hospital admission following outpatient surgery [5-7]. PONV can occur during the day after a surgical procedure or beyond [8]. In the first 24 hours postoperatively, the highest incidence of emetic sequelae is observed in patients undergoing laparoscopic gynecologic surgery or receiving general anesthesia [9,10]. Abdominal surgery is also a risk factor for PONV, with an incidence in excess of 50% [1]. The overall incidence of PONV after general anesthesia in outpatients has been reported to be 37%, although several factors, including sex, age, history of PONV, and opiate administration, influence the risk [11].

The serotonin 5-HT_3_ receptor antagonists (RAs) commonly are used for prophylaxis of PONV; however, there are fewer trials examining their use for treatment and rescue of PONV. Guidelines from the Society for Ambulatory Anesthesia (SAMBA) recommend that when PONV occurs after antiemetic prophylaxis, an agent from a different class should be used as rescue treatment [12]. Candiotti and colleagues investigated 88 women who developed PONV after ondansetron prophylaxis; these patients were randomly assigned to receive a repeat dose of ondansetron 4 mg, granisetron 1 mg, or granisetron 0.1 mg and were then followed for 24 hours [13]. The authors concluded that patients who failed ondansetron prophylaxis did not have a significant response to crossover administration of another 5-HT_3_ RA (ie, granisetron). In contrast, de Wit et al. demonstrated a benefit to rescue administration using granisetron and dexamethasone in patients receiving highly emetogenic chemotherapy who had failed ondansetron and dexamethasone prophylaxis [14]. Note that the former study evaluated PONV, while the latter assessed cancer-induced nausea and vomiting; differences may exist between these 2 populations. Thus, this issue remains unsettled.

Palonosetron is a pharmacologically distinct 5-HT_3_ RA with a greater binding affinity and longer half-life than older agents in this class [15]. Binding isotherms, equilibrium diagnostic tests, and kinetic diagnostic tests show that palonosetron is an allosteric antagonist with positive cooperativity, unlike ondansetron and granisetron. Differential effects on [^3^H]-ligand binding indicate that palonosetron interacts at different or additional sites on the 5-HT_3_ receptor compared with the binding profiles of granisetron or ondansetron. Unlike these agents, palonosetron also elicits 5-HT_3_ receptor internalization and promotes extended inhibition of receptor activity [16].

Two studies have shown that, compared with placebo, a single intravenous dose of palonosetron 0.075 mg effectively reduced emesis, nausea intensity, and rescue medication use in patients, particularly within 24 hours after surgery [17,18]. When directly compared with ondansetron before laparoscopic surgery or thyroidectomy, palonosetron showed similar or superior efficacy for prevention of PONV [19-23]; however, to our knowledge use of palonosetron as rescue therapy after ondansetron failure has not been assessed. The pharmacological profile of palonosetron, combined with its efficacy and favorable comparisons with ondansetron for the prevention of PONV, prompted the hypothesis that palonosetron may be effective as rescue therapy in patients for whom preoperative prophylaxis with another 5-HT_3_ RA had been unsuccessful, despite SAMBA recommendations to use an agent from another class [12]. The current phase II study evaluated the safety and efficacy of intravenous palonosetron 0.075 mg and intravenous ondansetron 4 mg (the currently approved doses) as rescue medications in patients experiencing PONV in the postanesthesia care unit (PACU) following unsuccessful prophylaxis with ondansetron.

## Methods

This was a randomized, open-label, multicenter trial that compared palonosetron with ondansetron using a 1:1 ratio as rescue therapy in outpatients who developed PONV in the PACU after receiving prophylactic ondansetron. Study objectives were to assess efficacy and safety of palonosetron and ondansetron when used in outpatients as rescue therapy for PONV in the PACU. The study was registered with ClinicalTrials.gov on August 27, 2009 (NCT00967499). Approval of the research protocol was required by each study center’s institutional review board/ethics committee prior to patient randomization, and the study complied with the Declaration of Helsinki and Good Clinical Practice guidelines (the International Conference on Harmonisation), with written informed consent obtained from all patients. This was an open-label study, and study drug preparation and dispensing were performed by the site pharmacist. A list of study sites and investigators is provided in Additional file 1.

### Patient selection

Outpatients undergoing elective laparoscopic abdominal or gynecological surgery who required general endotracheal anesthesia for ≥30 minutes were eligible for randomization if they met these criteria: ≥18 years of age, American Society of Anesthesiologists (ASA) physical status I to III, and ≥2 of the following PONV risk factors: female, nonsmoker, and history of PONV and/or currently prone to motion sickness. Exclusion criteria included chemotherapy within 4 weeks or emetogenic radiotherapy within 8 weeks of study entry; body mass index >40 kg/m^2^, use of investigational drugs within 30 days of study entry; use of drugs with potential antiemetic efficacy; or any nausea, vomiting, or retching within 24 hours prior to anesthesia.

### Treatment regimen and study design

On the day of surgery, patients received preoperative intravenous ondansetron 4 mg before induction of anesthesia per dosing and timing approved by the Food and Drug Administration (FDA). All patients had intravenous induction of general anesthesia per the standard of care at each site, were intubated, and received neuromuscular blockade, with reversal at the end of surgery as indicated. Regional or total intravenous anesthesia was not allowed. Using a minimization random allocation ratio, patients were randomized to rescue treatment with either intravenous palonosetron 0.075 mg or intravenous ondansetron 4 mg, both provided by Eisai Inc. Patients with symptoms requiring a rescue antiemetic—nausea score ≥4 on an 11-point Numeric Rating Scale (NRS), retching or vomiting, or patient request—within 6 hours of PACU admission were given the randomized drug within 10 minutes of investigator determination of necessity. The NRS was an 11-point linear scale on which patients rated their nausea, with 0 meaning no nausea and 10 meaning the worst possible nausea. These doses were selected because they correspond with those approved by the FDA for prevention of PONV and were anticipated to provide maximal effect.

Patients were assessed for their overall response to the rescue medication, and discharge from the PACU was not affected by study participation. Baseline assessments occurred at a screening visit within 2 weeks before the surgery (day 1), and efficacy and safety were evaluated at 0.5, 1, 2, 6, 12, 24, 48, and 72 hours after dosing with the rescue medication. After discharge from the PACU, all patients received a follow-up telephone call to review the patient diary, in which they were instructed to record emetic episodes, nausea severity (subjectively rated by patient) and duration, use of additional rescue drugs, and functioning related to nausea/emesis. In addition to baseline, the NRS was completed at the previously mentioned evaluation time points, with patients asked to rate the most severe nausea they had experienced since the last assessment.

The primary efficacy endpoint was the proportion of patients who achieved complete control (CC), defined as no emetic episode, no rescue medication, and a nausea severity score of ≤3 on the NRS, from 0 to 72 hours after rescue dosing. The percentage of patients with CC 0 to 30, 30 to 60, and 60 to 120 minutes also was assessed. Secondary efficacy endpoints were complete response (CR), defined as no emetic episode and no rescue medication; the proportions of patients with no emesis and no additional rescue medication in the 72 hours following the rescue dose; and the change from baseline nausea score using the NRS. Treatment-emergent adverse events (TEAEs), regardless of suspected causal relationship to the study medication, were recorded throughout.

### Statistical considerations

It was estimated that approximately 300 patients would need to be randomized to have 100 patients treated with either palonosetron or ondansetron; as this study was for proof of concept, no statistical justification of sample size was done. Patients were assigned to a treatment group using a minimization random allocation ratio. The primary efficacy parameter was analyzed using the full analysis set population, consisting of all patients who were randomized to and received study drug. Safety was evaluated for all randomized patients given rescue medication and who had ≥1 safety assessment after treatment.

Descriptive statistics were used for most primary and secondary efficacy parameters, as well as safety data. The Cochran-Mantel-Haenszel test was used to compare the CC rates between treatment groups, stratified to sex at a 2-sided significance level of 0.05. All secondary endpoints were analyzed using the same methods.

## Results

### Study disposition and baseline characteristics

**Figure 1:**
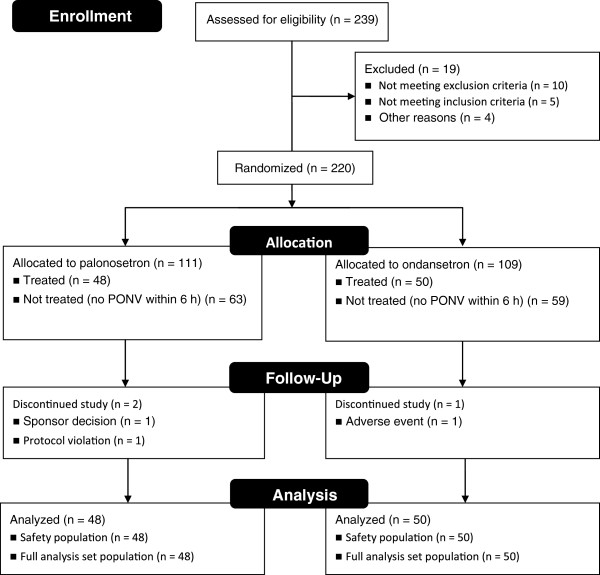
CONSORT flow diagram of patient disposition.

Patients were recruited from July 2009 to December 2009. Of 239 patients screened, 220 patients were randomized from 10 centers. See Figure 1 for complete patient disposition. In all, 98 patients experienced PONV that required rescue medication within 6 hours of PACU admission and were included in the study assessment. The patient demographics and baseline characteristics were similar in the palonosetron and ondansetron groups (Table 1). All patients were female, and most were nonsmokers. Most patients had low ASA scores (I or II).

**Table 1:** Baseline patient demographics and clinical characteristics

	**Palonosetron**	**Ondansetron**	**Total**
	**(n = 48)**	**(n = 50)**	**(N = 98)**
Age (y)			
Mean (SD)	41 (10.2)	43 (13.8)	42 (12.1)
Min, max	22, 62	21, 83	21, 83
Sex			
Female, n (%)	48 (100.0)	50 (100.0)	98 (100.0)
Race, n (%)			
White	36 (75.0)	36 (72.0)	72 (73.5)
Black or African American	7 (14.6)	9 (18.0)	16 (16.3)
Asian	1 (2.1)	3 (6.0)	4 (4.1)
Other	4 (8.3)	2 (4.0)	6 (6.1)
Nonsmoker*, n (%)	45 (93.8)	49 (98.0)	94 (95.9)
BMI (kg/m^2^)			
Mean (SD)	27.2 (5.4)	27.8 (5.0)	27.5 (5.2)
Min, max	17, 38	20, 39	17, 39
Duration of laparoscopic surgery (min)			
Mean (SD)	113.3 (69.4)	102.0 (59.8)	107.6 (64.6)
Median	92.5	81.5	88.5
Min, max	26, 306	34, 282	26, 306
Baseline NRS for nausea severity			
n	47	48	95
Mean (SD)	5.7 (1.84)	5.9 (1.86)	5.8 (1.84)
Min, max	2, 10	3, 10	2, 10
History of PONV^†^, n (%)	30 (62.5)	27 (54.0)	57 (58.2)
Type of laparoscopic surgery, n (%)			
Gynecological	37 (77.1)	31 (62.0)	68 (69.4)
Abdominal	11 (22.9)	19 (38.0)	30 (30.6)
ASA classification, n (%)			
I	20 (41.7)	20 (40.0)	40 (40.8)
II	25 (52.1)	24 (48.0)	49 (50.0)
III	3 (6.3)	6 (12.0)	9 (9.2)
Baseline opioid use, n (%)	47 (97.9)	50 (100.0)	97 (99.0)

*Abbreviations:ASA* American Society of Anesthesiologists, *BMI* body mass index, *NRS* Numeric Rating Scale, *PONV* postoperative nausea and vomiting, *SD* standard deviation.

*Never smoked or quit ≥12 months before participation in the study. ^†^History of PONV and/or currently prone to motion sickness.

### Complete control

As shown in Table 2, CC through 72 hours after dosing was achieved by 25.0% of palonosetron patients compared with 18.0% of ondansetron patients (95% confidence interval [CI], -9.2, 23.3; p = 0.40). Assessment of the initial 24 hours demonstrated a similar efficacy profile, while efficacy increased for both drugs during the 24- to 72-hour time period, with CC reached by a similar portion of patients in both groups.

**Table 2:** Complete control achieved—primary efficacy endpoint

	**Palonosetron (n = 48)**	**Ondansetron (n = 50)**	**Difference, %**	**p-value**
	**n (%)**	**n (%)**	**(95% CI)**	
0-72 h	12 (25.0)	9 (18.0)	7.0 (-9.2, 23.2)	0.40
0-24 h	12 (25.0)	10 (20.0)	5.0 (-11.5, 21.5)	0.56
24-72 h	40 (83.3)	40 (80.0)	3.3 (-12.0, 18.6)	0.67

*Abbreviation*: *CI* confidence interval.

A total of 56.3% of palonosetron patients and 58.0% of ondansetron patients achieved CC within the first 30 minutes of dosing. Across the 3 time periods examined during the first 120 minutes, CC ranged from 50.0% to 66.7% in the palonosetron group and from 58.0% to 62.0% in the ondansetron group.

### Secondary efficacy endpoints

The secondary endpoints of CR, no emesis, and no additional rescue medication are shown in Table 3 for the 0- to 72-, 0- to 24-, and 24- to 72-hour time periods. With the exception of a result that possibly favored palonosetron over ondansetron for no emesis over the entire 72-hour evaluation period (p = 0.057), none of the other comparisons approached statistical significance.

**Table 3:** Secondary efficacy endpoints

	**Palonosetron (n = 48)**	**Ondansetron (n = 50)**	**Difference,%**	**p-value**
			**(95%CI)**	
	**n (%)**	**n (%)**		
Complete response				
0-72 h	15 (31.3)	13 (26.0)	5.3 (-12.6, 23.1)	0.57
0-24 h	15 (31.3)	15 (30.0)	1.3 (-17.0, 19.5)	0.89
24-72 h	43 (89.6)	41 (82.0)	7.6 (-6.1, 21.3)	0.29
No emesis				
0-72 h	34 (70.8)	26 (52.0)	18.8 (-0.06, 37.7)	0.057
0-24 h	34 (70.8)	29 (58.0)	12.8 (-5.9, 31.6)	0.19
24-72 h	45 (93.8)	44 (88.0)	5.8 (-5.6, 17.1)	0.33
No additional rescue medication				
0-72 h	18 (37.5)	22 (44.0)	-6.5 (-25.9, 12.9)	0.52
0-24 h	18 (37.5)	23 (46.0)	-8.5 (-28.0, 11.0)	0.40
24-72 h	44 (91.7)	43 (86.0)	5.7 (-6.7, 18.1)	0.38

*Abbreviation:CI* confidence interval.

Prior to rescue medication administration, moderate nausea (as rated subjectively by patient) was experienced by 66.7% of patients in the palonosetron group and 62.0% of patients in the ondansetron group. After 24 hours no nausea was reported by 83.3% and 82.0% of patients treated with palonosetron and ondansetron, respectively, and at the end of 72 hours no nausea was recorded by 79.2% and 82.0% of palonosetron and ondansetron patients, respectively. Baseline NRS severity scores were similar for the 2 groups (5.7 and 5.9 in the palonosetron and ondansetron arms, respectively). Changes were most substantial in the 24- to 72-hour time period, with similar decreases in NRS scores, ranging from -5.1 to -5.3 and -5.3 to -5.6 in the palonosetron and ondansetron arms, respectively.

### Safety profile

The safety profiles for palonosetron and ondansetron were comparable, with a similar number of patients experiencing TEAEs during the 72-hour evaluation period (Table 4). Other than procedural pain, which was not substantially different between groups, the most common TEAEs in both groups were headache, constipation, and dizziness: 14.6%, 8.3%, and 6.3% and 12.0%, 10.0%, and 8.0% in the palonosetron and ondansetron groups, respectively. Most TEAEs were mild, but 6 palonosetron patients (12.5%) and 8 ondansetron patients (16.0%) experienced serious TEAEs, primarily gastrointestinal effects. No serious TEAEs were attributed to treatment with palonosetron, and 1 (2.0%) serious TEAE was thought to be related to ondansetron treatment.

**Table 4:** Treatment-emergent adverse events

	**Palonosetron**	**Ondansetron**	**Total**
	**(n = 48)**	**(n = 50)**	**(N = 98)**
TEAEs	43 (89.6)	49 (98.0)	92 (93.9)
Treatment-related AEs	5 (10.4)	4 (8.0)	9 (9.2)
Serious TEAEs	6 (12.5)	8 (16.0)	14 (14.3)
Treatment-related serious TEAEs	0 (0.0)	1 (2.0)	1 (1.0)
Deaths	0 (0.0)	0 (0.0)	0 (0.0)

*Abbreviations*: *AEs* adverse events, *TEAEs* treatment-emergent adverse events.

All data reported as n (%).

## Discussion

The use of prophylactic antiemetics is intended to prevent episodes of vomiting, eliminate or lessen the severity of nausea, and minimize or remove the need for PONV rescue medications. 5-HT_3_ RAs have proven effective for the prevention and treatment of PONV, with minimal adverse effects [12]. As a class, 5-HT_3_ RAs are generally safe at the usual doses used to prevent or treat PONV, with no dose-related sedation or extrapyramidal reactions and no significant effects on vital signs [24]. For prophylaxis of PONV, palonosetron has demonstrated efficacy similar to or superior to ondansetron [19-23]; however, they have not been compared for rescue after ondansetron failure. If patients who receive no antiemetic prophylaxis before surgery experience PONV, a 5-HT_3_ RA may be of benefit [12]. According to the SAMBA guidelines, if patients experience PONV after prophylaxis was given, an agent should be chosen from a therapeutic class different from the one administered prophylactically [12]. Because the mechanism of action and pharmacokinetics for palonosetron differ substantially from other 5-HT_3_ RAs, its use seemed reasonable when ondansetron had failed. Ondansetron rescue of ondansetron prophylaxis served as an active drug comparator with previously known results.

The primary endpoint of the study, proportion of patients achieving CC over the 72-hour evaluation period, was achieved in 25.0% and 18.0% of patients receiving PONV rescue treatment with palonosetron and ondansetron, respectively, showing no statistical difference (p = 0.40). The lack of statistical significance affirms an earlier study assessing granisetron rescue therapy following failed ondansetron prophylaxis [13]. Contrary to our estimate that 100 patients per treatment group would be appropriate, a post hoc power analysis showed that approximately 540 patients actually would be needed per arm to demonstrate a statistically significant difference in the primary endpoint with the results seen here (25.0% vs 18.0%). However, at the time, the study was carried out assuming a much larger treatment difference between palonosetron and ondansetron arms.

The 95% CI for the difference ranged from -9.2% (favoring ondansetron) to 23.2% (favoring palonosetron). Because the pharmacology of palonosetron differs from other 5-HT_3_ RAs, as described earlier, it seemed plausible that palonosetron might prove effective when another agent from the same class already has been used for PONV prophylaxis. If there is a difference, however, it would seem to be small based on this study. Differences in the 0- to 24-hour and 24- to 72-hour time periods also were not statistically significant.

Among the secondary outcomes of CR, no emesis, and no additional rescue medication, differences between the two arms showed a lack of statistical significance at all times. However, as with the primary endpoint, the 95% CI had a wide overlap across zero, and clinically relevant differences between palonosetron and ondansetron cannot be ruled out. Over the entire 72-hour assessment period, a lack of emesis was reported by 70.8% and 52.0% (95% CI -0.06, 37.7; p = 0.057) of palonosetron and ondansetron patients, respectively, showing a trend toward statistical significance, possibly due to the much longer half-life of palonosetron. Patient self-reported nausea showed similar improvements with palonosetron and ondansetron. The safety analyses demonstrated that both palonosetron and ondansetron were well tolerated, with no notable differences in safety parameters between groups.

Limitations of the current study include the lack of blinding, the timing of ondansetron prophylaxis, and the dosing of drugs. Drug dosing and time of administration needed to be consistent with FDA-approved labeling, so alternative methods of administration recommended by others could not be evaluated here. The most recent SAMBA guidelines recommend giving prophylactic ondansetron at the end of surgery, while administering palonosetron prior to surgery [12]. In this study, ondansetron was given at the induction of anesthesia, as per product labeling; however, this is not likely to have influenced rescue therapy. In addition, SAMBA guidelines recommend use of a different class of agent from the one given for PONV prophylaxis for PONV rescue; however, palonosetron was evaluated in this trial because of its unique pharmacological properties compared with other 5-HT_3_ RAs, as described earlier. The authors acknowledge that we could potentially learn more from a study designed with a different comparator than ondansetron (eg, an antiemetic with a different mechanism of action relative to the 5-HT_3_ RAs). In addition, although the study was open to both males and females, male patients were unable to be recruited. The incidence of PONV is higher in females, but the results cannot necessarily be extrapolated to males.

## Conclusions

Palonosetron and ondansetron did not show differences in the primary efficacy endpoint of CC during the 72 hours after study drug administration. At the earliest time points, there were no differences between palonosetron and ondansetron treatment, but in the full 0- to 72-hour period, there was a trend toward greater efficacy for palonosetron for patients experiencing no emesis after rescue, possibly because the longer half-life of palonosetron may make it more effective than ondansetron for delayed emesis. Outside of this trend toward less emesis overall, there does not appear to be a significant difference between palonosetron and ondansetron for rescue treatment of PONV after failure of ondansetron prophylaxis, supporting the SAMBA recommendations to use an antiemetic from another class.

## Abbreviations

5-HT_3_: Serotonin; ASA: American Society of Anesthesiologists; CC: Complete control; CI: Confidence interval; CR: Complete response; FDA: US Food and Drug Administration; PACU: Postanesthesia care unit; NRS: Numeric rating scale; PONV: Postoperative nausea and vomiting; RA: Receptor antagonist; SAMBA: Society for Ambulatory Anesthesia; SD: Standard deviation; TEAE: Treatment-emergent adverse event.

## Competing interests

KAC receives grant/research support, serves as a consultant to, and serves on the speakers bureau of Helsinn; SRA was an employee of Eisai Inc. at the time of the study; DC is a current employee of Eisai Inc.; TJG receives research/grant support from Eisai Inc. and NIKOM, and serves on the speakers’ bureaus of Baxter, Hospira, Pacira, and Fresenius.

## Authors’ contributions

KAC helped design and conduct the study, analyze the data, and write the manuscript. SRA helped design and coordinate the study. DC helped design and coordinate the study. TJG helped design the study. All authors reviewed drafts of the manuscript and provided final approval of the manuscript for submission.

## Pre-publication history

The pre-publication history for this paper can be accessed here:

http://www.biomedcentral.com/2050-6511/15/45/prepub

## Supplementary Material

Additional file 1 Study site investigators. Click here for file
