# Supplementary material for: Palonosetron versus ondansetron as rescue medication for postoperative nausea and vomiting: a randomized, multicenter, open-label study
Source: BMC Pharmacol Toxicol. 2014 Aug 16;15:45. doi: 10.1186/2050-6511-15-45 (PMC4152758; doi:10.1186/2050-6511-15-45)
Supplement: Additional file 1 — Study site investigators. [file 2050-6511-15-45-S1.docx]

**Additional File 1** **Study site investigators**

[File format: Word.docx]

| **Investigator** | **Institution** |
| --- | --- |
| Apfel, Christian | University of California San Francisco–Mt. Zion Hospital, San Francisco, CA |
| Bergese, Sergio D. | The Ohio State University Medical Center, Columbus, OH |
| Candiotti, Keith A. | University of Miami–Jackson Memorial Hospital, Miami, FL |
| Gan, Tong | Duke University Medical Center, Durham, NC |
| Jones, R. Kevin | Accurate Clinical Trials, Inc., Laguna Hills, CA |
| Kovac, Anthony L. | University of Kansas Medical Center, Kansas City, MO |
| Miller, Howard | Research Concepts, Ltd., Houston, TX |
| Minkowitz, Harold Sydney | Research Concepts, Ltd., and Memorial Hermann Memorial City Medical Center, Houston, TX |
| Roberson, Charles | Scott & White Memorial Hospital, Temple, TX |
| Wininger, Steven | Precision Trails, Phoenix, AZ |
